# Doctoral Studies as part of an Innovative Training Network (ITN): Early Stage Researcher (ESR) experiences

**DOI:** 10.12688/openreseurope.13094.1

**Published:** 2021-04-07

**Authors:** Roshni Biswas, Axel Schiller, Chiara Casolani, Elza Daoud, Albi Dode, Eleni Genitsaridi, Laure Jacquemin, Nuwan Liyanage, Matheus Lourenco, Punitkumar Makani, Vinay Parameshwarappa, Constanze Riha, Jose L Santacruz, Maryam Shabbir, Jorge Simoes, Natalia Trpchevska, Stefan Schoisswohl

**Affiliations:** 1 European School on Interdisciplinary Tinnitus Research (ESIT), Regensburg, Germany; 2 Hearing Sciences, Mental Health and Clinical Neurosciences, School of Medicine, University of Nottingham, Nottingham, UK; 3 Department of Environmental Health Sciences, Istituto di Ricerche Farmacologiche Mario Negri IRCCS, Milan, Italy; 4 Department of Psychiatry and Psychotherapy, University of Regensburg, Regensburg, Germany; 5 Tinnitus Assessment Causes Treatments (TIN-ACT), Groningen, The Netherlands; 6 Hearing System Section, Department of Health Technology, Technical University of Denmark, Lyngby, Denmark; 7 Oticon A/S, DK-2765 Smørum, Denmark; 8 Interacoustics Research Unit, DK-2800 Lyngby, Denmark; 9 Centre National de la Recherche Scientifique, Aix-Marseille University, Marseille, France; 10 Institute of Databases and Information Systems, University of Ulm, Ulm, Germany; 11 NIHR Nottingham Biomedical Research Centre, Nottingham, UK; 12 Department of Otorhinolaryngology and Head and Neck Surgery, Antwerp University Hospital, Edegem, Belgium; 13 Dept. of Translational Neurosciences, Faculty of Medicine and Health Sciences, University of Antwerp, Wilrijk, Belgium; 14 University of Zurich, Zurich, Switzerland; 15 Department of Otorhinolaryngology, Head and Neck Surgery, University Hospital Zurich, Zurich, Switzerland; 16 Experimental Health Psychology, Faculty of Psychology and Neuroscience, Maastricht University, Maastricht, The Netherlands; 17 Health Psychology Research Group, Faculty of Psychology and Educational Sciences,, University of Leuven, Leuven, Belgium; 18 Department of Otorhinolaryngology, Head and Neck Surgery, University of Groningen, University Medical Center Groningen, Groningen, The Netherlands; 19 Department of Neuropsychology, University of Zurich, Zurich, Switzerland; 20 Graduate School of Medical Sciences (Research School of Behavioral and Cognitive Neurosciences), University of Groningen, Groningen, The Netherlands; 21 Experimental Audiology, Department of Physiology and Pharmacology, Biomedicum, Karolinska Institutet, Stockholm, Sweden

**Keywords:** Innovative Training Network, PhD, doctoral training, PhD experience, European Union

## Abstract

**Background: **The Marie-Skłodowska-Curie Actions’ (MSCA) Innovative Training Network (ITN) is a doctoral training programme jointly implemented by academic institutions and industries from countries across Europe and beyond. To our knowledge no study has examined the experience of students participating in MSCA-ITNs. This study aims to evaluate and report MSCA-ITN Early Stage Researcher (ESR) experiences.

**Methods: **The Innovative Training Network - Evaluation Questionnaire (ITN-EQ) was developed to assess supervision, training, collaborations and experiences of ESRs and forwarded to two tinnitus-related ITNs and seven ITNs of other disciplines.

**Results: **Key advantages identified included better career prospects, multidisciplinary research opportunities/ collaborations, international exposure, personal/ professional development, plus generous salaries and research budgets. However, lack of a common EU framework resulted in the experience being largely dependent on host institution, country and supervisor. Moreover, managing the dual requirements of ITNs and host institutions while completing a three-year PhD seemed challenging for most ESRs. ESR involvement in workshop and training school planning was desirable. More than 80% of ESRs rated the overall ITN experience favourably and 98.3% would recommend the same to prospective PhD students.

**Conclusions: **This report could provide valuable insights in planning and management of future ITNs and could assist prospective students in their decision of joining an ITN for their PhD.

## Plain language summary

The Marie-Skłodowska-Curie Actions’ (MSCA) Innovative Training Network (ITN), funded by the European Union (EU), is a doctoral training programme jointly implemented by academic institutions and industries from countries across Europe and beyond. To our knowledge no study has systematically examined the experience of students participating in MSCA-ITNs. This study aims to evaluate and report MSCA-ITN ESRs’ experiences. To assess supervision, training, international collaborations and experiences of ESRs, we developed the Innovative Training Network - Evaluation Questionnaire (ITN-EQ). The ITN-EQ, consisting of 46 questions, was forwarded via an online survey tool to two tinnitus-related ITNs, and seven other ITNs related to diverse disciplines. Key advantages identified by ESRs included better career prospects, opportunities for multidisciplinary research and collaborations, scope for international exposure, personal and professional development, plus a generous salary and research budget. However, lack of a common EU framework resulted in the experience being largely dependent on host institution, country and supervisor. Moreover, managing the dual requirements of ITNs and host institutions while completing a three-year PhD seemed challenging for most ESRs. ESR involvement in planning of workshops and training schools was desirable. More than 80% of ESRs rated the overall ITN experience favourably. 92% of them would still choose doing a PhD as part of an ITN and 98.3% would recommend the same to prospective PhD-students. This report is a novel attempt that could provide valuable insights in planning and management of future ITNs and could assist prospective students in their decision of joining an ITN for their PhD.

## Introduction

Training of doctoral students is an important part of the academic commitment of most tertiary institutions, and a doctorate degree is perceived as the pinnacle of academic achievement ( Millett & Nettles, 2006; Park, 2005). In recent years, doctoral training programmes have become more structured with academic institutions opting to improve the training experiences of students by accommodating doctoral research projects within a defined training framework ( Doonan *et al.*, 2018). The European Union’s (EU) Innovative training network (ITN) under the Marie Skłodowska-Curie Actions (MSCA) is an example of a multinational interdisciplinary PhD programme for training new generations of researchers.

A major objective of the European Commission (EC) is to promote innovation and sustainability through cutting-edge research in its member states and affiliated countries i.e., the European Research Area. Horizon 2020, set out to cover the period of 2014 to 2020, is a central instrument of the EC’s eighth strategic funding programme in the area of innovation, research and technological development. The aims of Horizon 2020 are grouped under three pillars, “Excellent Science”, “Industrial Leadership”, and “Societal Challenges”, with an overall budget estimated at around 80 billion euros.

Under the umbrella of Horizon 2020, the MSCA represents a dedicated programme for supporting international and interdisciplinary education and mobility throughout Europe, promoting research careers in all scientific fields by providing fellowships to young academics from all over the world. MSCA offers several programmes of research support. The two main means of funding intended for early career or Early Stage Researchers (ESRs) are International Training Networks (ITNs) and Individual Fellowships, depending on the current educational and career stages of an individual. Individual Fellowships are directed towards individual postdoctoral researchers after the completion of a PhD. In this paper, we discuss the MSCA-ITNs which aim to provide PhD training. The MSCA-ITN has a unique structure where multiple partner organisations including universities, research institutions, research infrastructures, businesses, small to medium enterprises, and other establishments from different countries across Europe and beyond, jointly build a consortium for a structured doctoral training programme in any specific research topic.

The European School on Interdisciplinary Tinnitus Research (ESIT) and Tinnitus Assessment Causes Treatments (TIN-ACT), are two such ITNs. The research topic for these two ITNs is tinnitus, which is the auditory perception of a ringing or buzzing sound in the absence of any corresponding external stimuli ( Langguth *et al.*, 2013). Tinnitus is a relatively common condition affecting approximately 15% of the adult population with neither a cure nor sufficient evidence for a single effective treatment ( Baguley *et al.*, 2013). Heterogeneity with regards to perception, distress, aetiology and treatment response are some of the major challenges in tinnitus research ( Cederroth *et al.*, 2019). Therefore, an interdisciplinary approach seems to be the way forward to address the unmet needs in this field. Both ESIT and TIN-ACT with their inherent multidisciplinary structure, provide appropriate platforms for this approach.

Funded by the MSCA, ESIT represents the first doctoral training program specialized in tinnitus research. It is a collaboration of experts from relevant fields such as epidemiology, psychology, animal research, genetics and statistics, at 12 research institutions across 10 countries of the EU, the United Kingdom and Switzerland. ESIT aims at tackling the current issues and challenges in tinnitus research by conducting high quality and ground-breaking interdisciplinary science. Within this framework, a new generation of young tinnitus researchers are trained. Apart from being educated in tinnitus-related science, the 15 PhD students undergo training in transferable and soft skills, as well as participating in academic and industrial internships. Within three work packages (WP) 15 individual PhD-projects are conducted which focus on: 1) tinnitus heterogeneity, 2) personalized treatment solutions and 3) comparability of research findings (see *Extended data* Table 1 ( Biswas *et al.*, 2021)). Numerous scientific publications have emerged from the PhD projects within the ESIT and TINACT consortia.

An additional objective of ESIT was to establish a multidisciplinary network for ESRs, and promote interdisciplinary collaborations which is apparent from the several joint publications with more planned projects on the way ( Genitsaridi *et al.,* 2019; Mehdi *et al.*, 2020a; Mehdi *et al.*, 2020b; Schlee *et al.*, 2020; Schoisswohl *et al.*, 2019; Simoes *et al.*, 2019; Spencer *et al.*, 2019). A more detailed description of the ESIT project has been reported in an article by Schlee *et al.* ( Schlee *et al.*, 2018).

TIN-ACT, a second MSCA-funded doctoral training program, was formed following ESIT and it also focuses on tinnitus research. TIN-ACT comprises 15 ESRs across seven universities, three companies, patient associations and training institutions across six countries, with projects addressing challenges in the following domains of tinnitus research: assessments, causes and treatments. Overall, there are six WPs in TIN-ACT, of which three address training (WP1), management (WP6) and dissemination (WP5) while the remaining three (WP2, WP3 and WP4) address the research fields in which the projects are divided across. TIN-ACT ESRs are also required to undergo various training to develop research-specialist and job-specific skills along with other complimentary soft skills. The ESRs are also expected to complete two secondments- one at a partner academic institution and one at an industrial partner. The 15 PhD projects fall under the following three work packages: WP2- Assessments, WP3- Causes, and WP4- Treatments (for a project overview see *Extended data* Table 2 ( Biswas *et al.*, 2021)).

## Objective

The PhD journey as an ESR is distinctive from others programmes, as doctoral students here are expected to fulfil dual requirements - as part of their host universities, and also as part of their respective ITN. They are trained alongside peers who investigate different aspects of a common research topic. Moreover, there are inherent structural and organisational attributes of the MSCA-ITN that shape the ESRs’ experience.

There are three main objectives of this report. Firstly, to evaluate doctoral programmes as part of a tinnitus-related MSCA-ITN. Secondly, to explore the ESR experiences from a number of other MSCA-ITNs from various disciplines in order to understand the agreement between the perspectives of ESRs from tinnitus-related and unrelated ITNs. Lastly, to evaluate the experience of individual PhD candidates who participated in MSCA-ITN training activities and compare them with the ESR experience.

## Methods

### Survey development and administration

In order to identify important topics concerning ESRs’ experiences, ESRs (TIN-ACT and ESIT) were invited to voluntary participate in an unstructured group session/ interview moderated by authors RB, AS and SS. The aim was to identify some broad domains that could potentially impact their PhD journey. Additional relevant domains were identified using an existing questionnaire/survey that addressed various aspects of training and research experiences of EU-funded researchers.

A set of questions and response options was then formulated that dealt with the identified topics. In a next step, three individual researchers with knowledge in the field of questionnaire development reviewed the created set of questions and response options. Although, not directly part of the ITN, these three researchers, who are involved in doctoral training in various capacities, were particularly selected given their involvement in ITN-related training schools and workshops and awareness of the MSCA-ITN structure. Based on these steps, the Innovative Training Network – Experience Questionnaire (ITN-EQ) was designed. It included 46 questions to address the following aspects of ESRs experiences: general information/ demographics; studying/ living abroad; supervision, budget, training and workshops; conferences; collaborations; secondments; overall ITN experiences. Domains, questions and corresponding response options – open-ended, closed-ended, multiple-choice, 10-point numeric rating scale (NRS), five-point Likert scale, are reported in *Extended data* Tables 3 and 4 ( Biswas *et al.*, 2021). Given that the questionnaires were created during a time when all fields of life were affected by the Covid-19 pandemic, an additional question was developed to assess how this crisis affected ESRs and individual PhD students’ progress, research experiments and/ or secondments. The target group for this survey was ESRs who were already in the second or third year of their PhD and therefore had experienced various aspects of the MSCA-ITN doctoral training model.

To compare the experiences of PhDs as part of an ITN with that of individual PhDs the ITN-EQ was adapted to the modified ITN-EQ (mITN-EQ which targeted individual PhDs who were associated with ITN programs through attendance in various workshops or training schools (details in *Extended data* Table 5 ( Biswas *et al.*, 2021)). For the actual survey, the ITN-EQ as well as the mITN-EQ were implemented in SurveyMonkey- an online survey platform -and was provided via a link to the respective ITN management team to circulate within their PhD consortium for potential participants between May and June 2020.

### Survey participants

28 ESRs from ESIT and TIN-ACT ITNs were invited by the respective ITN management teams to take part in the survey. Authors RB and SS (ESIT) were involved in the development of the questionnaires and therefore did not participate in the survey. For a wider MSCA-ITN perspective, ITN management teams were contacted with the request to circulate the survey within their respective consortia. The managing bodies of selected ITNs (with research topics in health, technology, basic sciences and humanities and having students in second and third year of PhD) were contacted for this purpose. Apart from the ITNs of which the authors were aware of, the Community Research and Development Information Service (CORDIS)- a directory of all EU-funded research initiatives, was used to search for other ITNs that met the eligibility criteria. 34 MSCA-ITNs were contacted, of which seven circulated the survey within their ITN consortia. All of the contacted ITNs were further requested to circulate the mITN-EQ within their network as well.

### Ethics statement

The survey was completely anonymous and did not require any registration or declaration of personal information like name, date of birth or e-mail contact details (no possibility to identify the respondents). Participants were ESRs who wanted to voluntary share their opinion and thoughts on their ESR experience. Thus, ethical approval was not sought for this study. Ethically relevant information about the current survey/ survey procedure were provided in accordance with the H2020 ethics self-assessment. Participants were informed about the aim of the survey and consented through their voluntary participation (conclusive consent).

### Data analysis

This was a descriptive study, where depending on the answer format, the results are represented as absolute/ relative numbers, descriptive statistics or text. Figures were used from the output of the online survey tool as well as created with the statistics software R (R version 4.0.2; R Foundation for Statistical Computing, Austria) and further improved via Adobe Illustrator CC 2020 (Adobe Inc., USA).

## Results

### Respondent characteristics

Within tinnitus-related ITNs, 24 ESRs (14 females; one preferred to not answer) aged between 24 and 35 years (M = 29.0, SD = 3.0) completed the survey. The majority reported to have an academic background in information sciences and engineering (45.8%). Within ITNs from other fields, the survey was completed by 58 ESRs, made up of 50% women and 50% men (three preferred not to answer, one missing). The ESRs were between the ages of 23 and 33 (M = 28.5, SD = 2.3) years and more than half of them reported to have an academic background in basic sciences (53.4%). Nine individual PhD-students (six females; one preferred not to answer) associated with nine MSCA-ITN programmes participated in the mITN-EQ. Individual PhD-students were between 23 and 31 years (M = 29.6, SD = 5.0) old and most of them declared to have an academic background in basic sciences (55.6%). Detailed information about the sample characteristics of ESRs from tinnitus-related ITNs ( *Extended data* Table 3), ITNs from other fields ( *Extended data* Table 4) as well as individual PhDs *(Extended data* Table 5) are provided as *Extended data* ( Biswas *et al.*, 2021).

### Evaluation of tinnitus related ITNs

**Figure 1.**
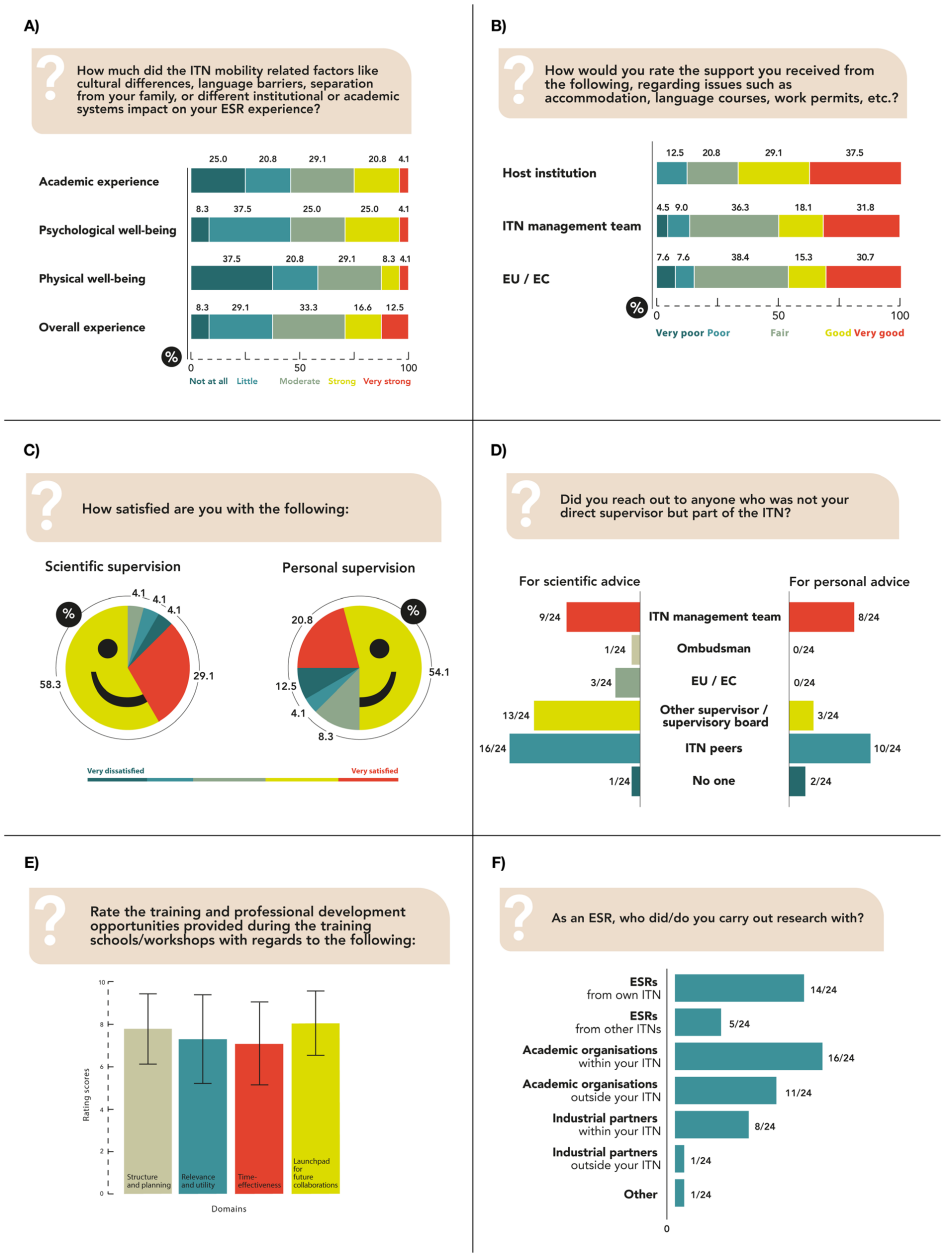
Tinnitus-related ESRs. ITN-EQ results of questions 9 ( **A**), 10 ( **B**), 12 and 13 ( **C**), 15 ( **D**), 19 ( **E**; error bars respresent standard deviations) and 26 ( **F**). ESR, Early Stage Researcher; ITN, Innovative Training Network; ITN-EQ, Innovative Training Network - Evaluation Questionnaire; EU, European Union; EC, European Commission.

Among 24 ESRs from ESIT and TIN-ACT who responded to the ITN-EQ, 12.5% required a visa for joining the program, 4.2% for participating in a conference, and 37.5% for both. Host institutions and ITN management teams helped ESRs in obtaining the required travel documents. 70.8% of participants had international experience prior to joining the MSCA-ITN, and in most cases for academic pursuits. Figure 1A shows the impact of ITN-related mobility on the respondents’ academic performance, physical, psychological and overall well-being, with most reporting little to moderate impact. Host institutions and supervisors, ITN management team and EU project team supported ESRs with accommodation, language courses and work permits ( Figure 1B). 62.5% were fully integrated and 25.0% moderately integrated to the host institution. Most ESRs were satisfied with the scientific and personal supervision experience ( Figure 1C). 83.3% ESRs benefited from the multidisciplinary ITN consortia, and 12 ESRs elaborated the advantages or disadvantages. Collaborative research, better learning opportunities, and an interdisciplinary approach to tinnitus helped ESRs develop a well-rounded perspective. 16.7% of ESRs with distinctive research fields did not find the multidisciplinary approach advantageous either due to a lack of common research interests or lack of supervisors with specific expertise. Although the ITN consortium comprises several stakeholders, ESRs mostly reached out either to their peers, other supervisors, or the ITN management teams, for both scientific and personal advice ( Figure 1D).

91.7% felt an initial settling allowance would be helpful. The overall funding budget was reported to be sufficient for both salary and research expenditures. The training schools and workshops experiences were consistently positive ( Figure 1E), and ITN experience was considered as very useful for personal development. Some suggested recommendations were: having fewer workshops, allowing more time for personal PhD projects, training on personal development as well as scientific topics, modifying the content of training modules to align with the scientific backgrounds and PhD progress of ESRs, providing preparatory materials for complex topics, and involving ESRs in planning of training activities. On average, ESRs attended 4.0 (SD = 3.1) conferences with 2.4 (SD = 2.3) oral or poster presentations in 2.4 (SD = 2.3), and 1.4 (SD = 2.1) conferences outside their field of specialisation. 75.0% of respondents had no experience in planning or execution of meetings or conferences. 83.3% were satisfied with their collaborations, which were mainly with other ESRs and academic organizations within their own ITN ( Figure 1F).

21.7% of ESRs had secondments in academic organisations, 8.7% in industry and 56.5% in both types of organisations. These secondments helped in gaining perspective on the workings of different types of institutions and influenced choice of academia or industry for future careers for 17.4% of respondents. However, for 82.6% of respondents this experience had no impact on the choice of future work sector. 91.3% of ESRs agreed that the ITN improved their career prospects. 52.2% of ESRs felt that being part of an ITN made the PhD journey easier, for 13.0% the ITN made the PhD more difficult, and for 34.8% it did not affect the PhD journey. However, 95.7% of ESRs would do their PhD as part of an ITN again and 23 of them (one missing value) would recommend the ITN to prospective PhD students. The main reported advantages of participating in an ITN included better career prospects, the opportunities for multidisciplinary research training and collaboration, scope for travel and exposure to different places and cultures, multiple sources of support in case of trouble with their research team/supervisor, development of personal and transferable skills through workshops, and a generous budget for salary and research support. Additionally, ESRs could reach out to peers in similar situations to discuss academic/ personal problems and relieve distress. However, lack of a common EU framework, resulted in the ITN experience being largely dependent on the local structure of the host country and institute. Time constraints from finishing the PhD in three years with no scope of extension in a demanding ITN setting and lack of local support in the presence of differences with the host institution and/or supervisor, made the ITN experience challenging. Additionally, traveling and associated paperwork (like visa processes), non-flexibility of secondments and workshops in terms of logistics and time, and ITN-related administrative responsibilities such as budget and financial paperwork, took away valuable time from actual research. Nonetheless, all ESRs rated the overall ITN experience either as “very good” or “good” ( Figure 3A). Notably, most ESRs had no prior idea of what the ITN entailed. Some ESRs anticipated traveling and opportunities for international training and life experiences, networking and learning, while some others expected a regular PhD experience having more solitary work. For all 23 ESRs (one missing value) the experience either met or exceeded their expectations. Detailed results can be seen in *Extended data* Table 4 ( Biswas *et al.*, 2021).

### Evaluation of ITNs related to other fields

**Figure 2.**
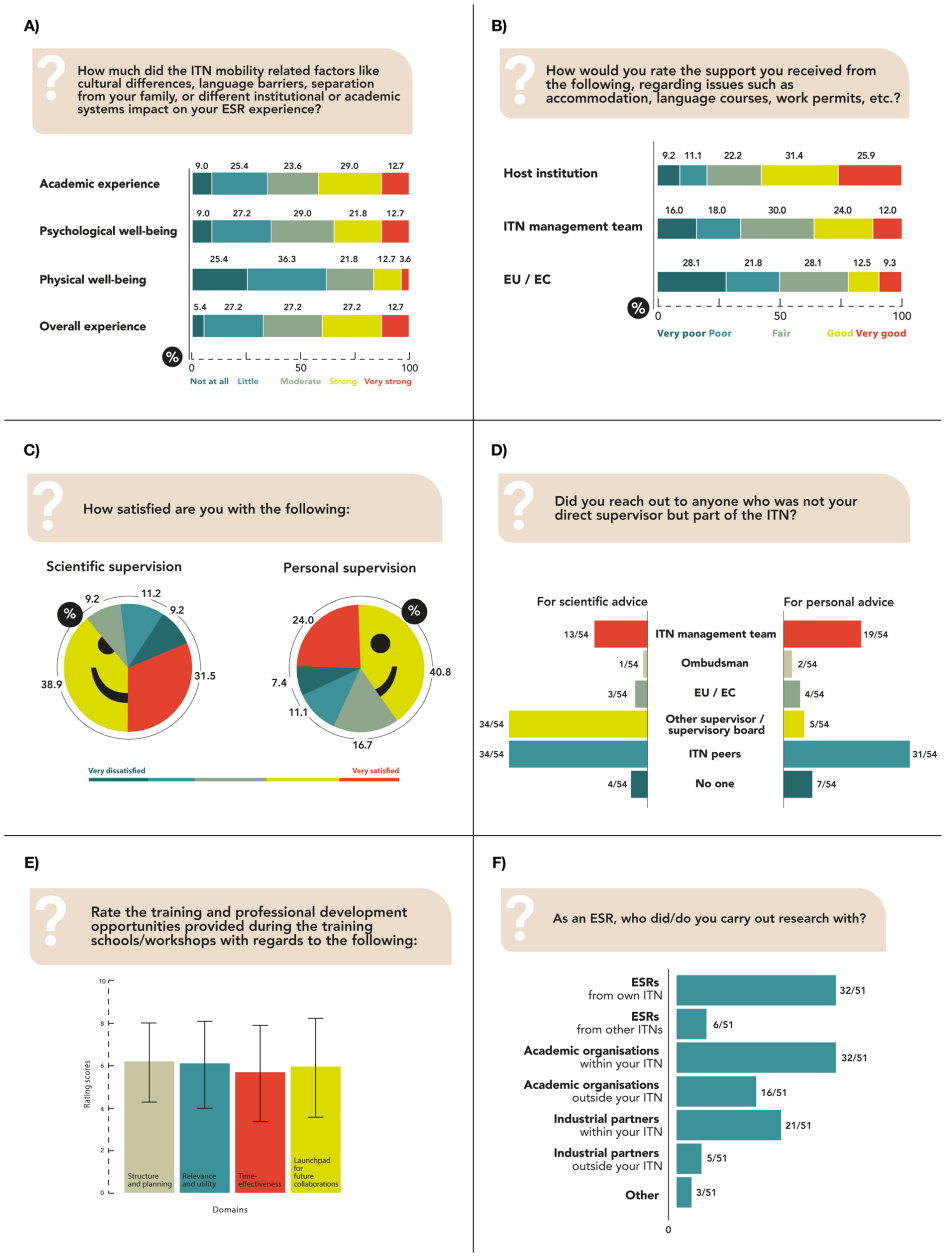
ESRs from other research disciplines. ITN-EQ results of questions 9 ( **A**), 10 ( **B**), 12 and 13 ( **C**), 15 ( **D**), 19 ( **E**; error bars respresent standard deviation) and 26 ( **F**). ESR, Early Stage Researcher; ITN, Innovative Training Network; ITN-EQ, Innovative Training Network - Evaluation Questionnaire; EU, European Union; EC, European Commission.

Among the 58 participants, 14.0% required a visa or travel document only to join the program, 5.3% of respondents only to attend conferences, while 66.7% ESRs required neither. Most ESRs reached out to host institution and ITN management teams for help with visa and travel documents, and were generally satisfied with the response ( Figure 2B). 63.2% respondents had prior experience of living abroad during which the majority was involved in various academic pursuits (previous degree, exchange students, summer school). Figure 2A illustrates the effect of mobility-related factors on academic experience, and physical, psychological and overall well-being. Support to settle in the new country came mostly from host institutions and were deemed as favourable by ESRs, 76.4% of whom felt mostly to fully integrated into the host team. 70.4% ESRs were either very satisfied or satisfied with the scientific supervision while 64.8% ESRs were either very satisfied or satisfied with the personal supervision received ( Figure 2C). 81.5% ESRs benefited from the multidisciplinary team of supervisors with increased possibilities of collaborative research, improved scientific skills and knowledge, gaining a platform for interdisciplinary approach to develop a well-rounded perspective, and additional support when supervisors could not help. 18.5% ESRs either did not need to collaborate because of a distinct research topic, or could not collaborate due to difficulties with the host institution and supervisor. Among the ITN stakeholders, ESRs mainly reached out to their peers and ITN management team for personal advice; for scientific advice they likewise reached out to their peers and also to other supervisors ( Figure 2D).

**Figure 3.**
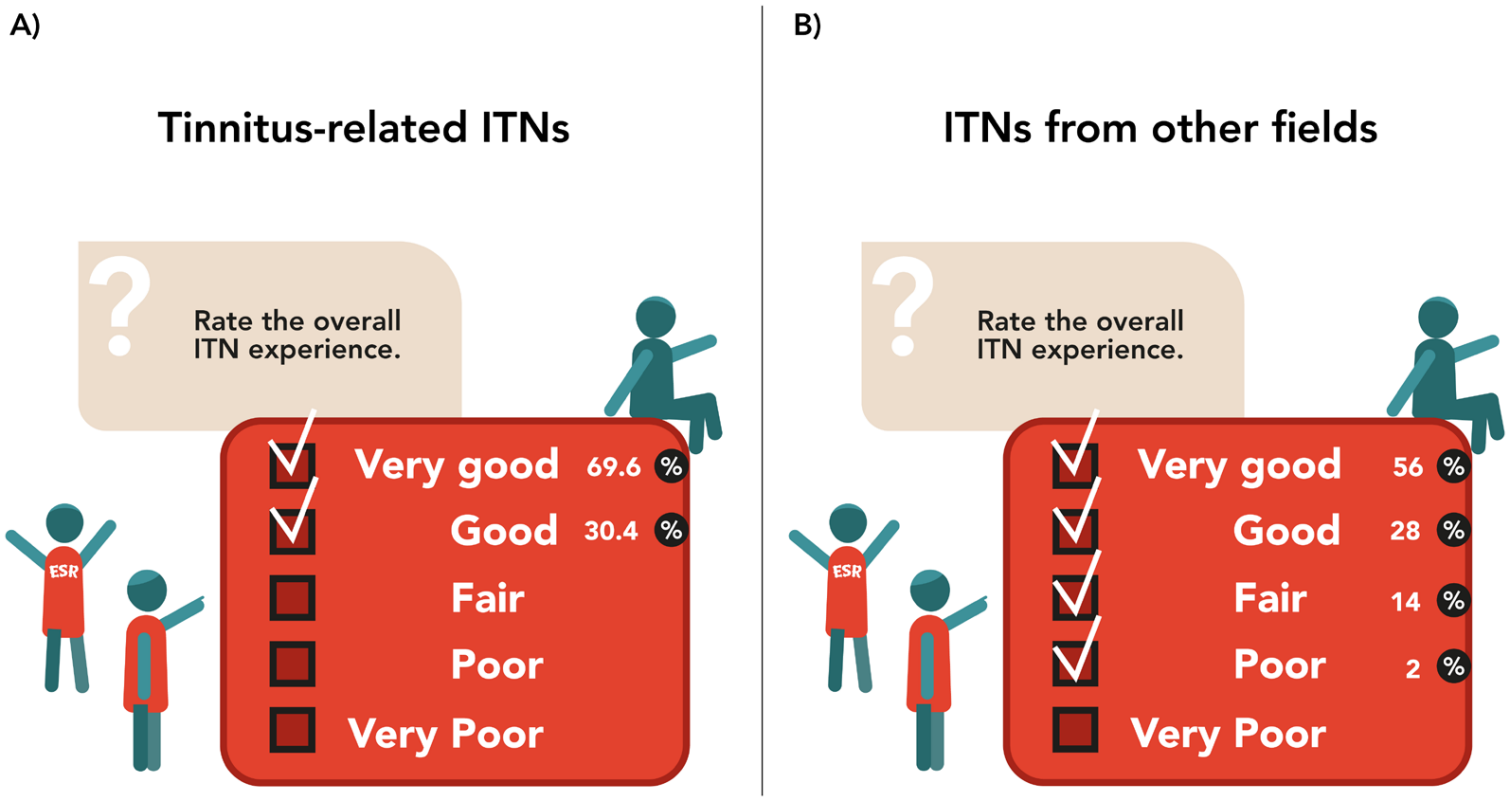
Overall ITN experience. ITN-EQ results of questions 36 for ESRs from tinnitus-related ITNs ( **A**) and from other fields ( **B**). ESR, Early Stage Researcher; ITN, Innovative Training Network; ITN-EQ, Innovative Training Network - Evaluation Questionnaire.

88.7% of ESRs agreed that an initial settling allowance would be helpful. Both the salary (M = 8.1, SD = 1.9) and research support (M = 8.6, SD = 1.8) were highly rated on a 1-10 numeric scale, and the institution specific reimbursement processes were satisfactory for 75.5% ESRs. The overall training schools and workshops experience was generally positive ( Figure 2E), and the majority of respondents (94.2%) reported the ITN experience to be useful for their personal development. Better organization of workshops, methodological training at PhD beginning, outreach activities nearer to the end, discussions on careers outside academia, PhD progress support, efficient time management, including ESRs in workshop planning, more networking time, and credits from the European Credit Transfer and Accumulation System (ECTS) for participation were some suggested recommendations. On average, ESRs attended 3.8 (SD = 2.7) conferences, with oral or poster presentations in 2.8 (SD = 2.2), and 1 (SD = 1.6) conferences outside their field of specialization. 57.7% respondents had no experience in planning and execution of meetings or conferences. 76.5% were “satisfied” to “very satisfied” with their collaborations, which were mainly with other ESRs and academic organizations within their own ITN ( Figure 2F). 21.6% were “neither satisfied nor dissatisfied” and 1.9% “dissatisfied” with the collaborations.

41.2% ESRs had secondments in academic organizations, 17.6% in industry and 41.2% in both types of organizations. For 37.3% respondents these secondments influenced their choice of academia or industry such as by helping in gaining perspective on the workings of different types of institutions. 29.4% ESRs either had no secondment or only in one type of organization, and therefore, the experience had no effect on their choice of work sector. 33.3% reported that it did not matter for them, since they already had previous exposures, the secondment was too short, they continued working on their regular projects at a different location or believed the experience was institution specific. 94.1% ESRs agreed that the ITN improved their career prospects given that it’s a prestigious opportunity in multidisciplinary research which enhanced personal development and assisted future career plans. Those who disagreed expressed that the experience was host country dependent and thus, not everyone offered adequate support to complete an international PhD as part of an ITN. 62.0% of ESRs felt that being a part of an ITN made the PhD journey easier, for 14.0% the ITN made the PhD more difficult, and for 24.0% it did not affect the PhD journey. However, 88.0% of respondents would do their PhD as part of an ITN again and 96% would recommend the ITN experience for prospective PhD students. The best experiences and main advantages of being a part of an ITN included better career prospects, personal and professional development, international experience in training and living abroad, interacting with multidisciplinary experts, a generous salary and research budget, exposure to both academia and industry and peer support from the consortia. Time constraints of a three year PhD in the demanding ITN setting, discrepancy of regulation between EU and host organization, insufficient scientific or supervision experience, time consuming trainings, organizing secondments, excessive traveling, personal and professional differences with the host institution and/ or supervisor, lack of integration into host or secondment institutions, as well as a large amount of bureaucratic necessities and documentation were some of the challenges that the ESRs faced. 84% respondents rated the overall ITN experience either as “very good” or “good” ( Figure 3B). Equally to ESRs from tinnitus-related ITNs, many were unaware of what the ITN experience entailed. Some ESRs anticipated travel and international experiences, exposure to academia and industry, networking and learning opportunities, and appreciated the better salary and research support, while some expected an experience like individual PhDs. For 76% of ESRs the ITN experience met or exceeded their expectations. *Extended data* Table 4 contains detailed results for ESRs from other ITNs ( Biswas *et al.*, 2021).

### Individual PhD candidates with exposure to ITN training

50% of the respondents travelled abroad for their PhD studies, while 50% did it locally. 87.5% reported full integration into their host institution, while 12.5% were slightly integrated. 37.5% of the respondents required visas or travel documents to join the program, 25% to attend conferences, and 50% for neither. 57.1% of the respondents were very satisfied with the scientific supervision, 14.3% were neither satisfied nor dissatisfied, and 28.6% were very dissatisfied. 42.9% respondents were very satisfied with the personal supervision, 28.6% were satisfied, 14.3% were neither satisfied nor dissatisfied, and 14.3% were dissatisfied (two missing). 28.6% found it difficult to reach out to experts from other fields for their research project, 57.1% found no difficulty and 14.3% did not reach out. On a numeric scale (1–10), the salary was rated as M = 5.4 (SD = 3.3) while research support was rated M = 4.6 (SD = 3.5). 57.2% students were “dissatisfied” to “very dissatisfied” with the local reimbursement processes while 42.9% were very satisfied. Individual PhDs attended M = 4.5 (SD = 2.9) ITN associated training schools or workshops. Structure and planning of ITN trainings were evaluated as M = 4.5 (SD = 3.2, Min = 1.0, Max = 8.0), relevance and utility as M = 4.2 (SD = 2.8, Min = 1.0, Max = 8.0), time-effectiveness as M = 4.7 (SD = 3.0, Min= 1.0, Max = 8.0), and launchpad for future collaborations as M = 4.3 (SD = 3.7, Min= 1.0, Max = 10.0). On average, students attended M = 5.0 (SD = 4.5) conferences, with oral or poster presentations at M = 4.2 (SD = 3.7) conferences within, and M = 1.2 (SD = 1.0) conferences outside their field of specialization. 83.3% respondents had no experience in planning and execution of meetings or conferences. 66.7% were very satisfied or satisfied with their collaborations, which were mainly with PhD candidates from their home institution.

66.7% respondents reported the ITN training schools as useful for personal development. More scientific coursework and rotating location of training schools were recommended changes. Networking opportunities and non-academic insights provided at training schools were appreciated, although inefficient organization and planning of training schools, and repetitive content were some of the concerns. One third of the respondents rated the overall ITN as very good, one third as good and the other third as fair. 80.0% respondents reported that participating in an ITN would have made the PhD journey more difficult. 40% respondents would recommend an ITN to prospective PhDs, 20% would not do so, and for the rest it would make no difference from an individual PhD. The individual PhD students noted travel, international experience, and networking opportunities as advantages of the ITN programs, while they observed that the experience depends on the organisational ability of the ITN management team and also that the large number of deliverables and activities make the ITN experience very demanding. Detailed evaluation results from individual PhDs can be found in *Extended data* Table 5 ( Biswas *et al.*, 2021).

### Covid-19 related issues

Covid-19 affected the PhD progress of 21 out of 24 ESRs from ESIT and TIN-ACT, 45 out of 58 from other ITNs and four out of nine individual PhD students who participated in this study. Difficulties in laboratory work, participant recruitment and data collection, modification of planned projects and cancellation of planned secondments were the major concerns. Situational anxiety, loss of productivity, uncertainty about projects and potential funding issues after the three years of ITN, along with general anxiety about friends and families abroad particularly affected the ESRs. For a detailed overview of doctoral students´ answers please see the respective tables in the *Extended data* ( Biswas *et al.*, 2021).

## Discussion

The aim of the current study was to evaluate the perspective of ESRs who participated in MSCA-ITNs related to tinnitus or other fields. To the best of our knowledge, this is the first attempt at gaining a student perspective on EU-funded consortia-based PhD programmes. Furthermore, we not only compared the perspectives of ESRs from tinnitus-related ITNs with those of ESRs from other fields on a descriptive level, but also with the experiences of students undertaking individual PhDs who participated in various ITN training activities. In the following section, the findings from the ESRs’ perspectives from both tinnitus and other fields are critically discussed.

The tinnitus-related ITNs included a more global student body with more than half the candidates requiring a visa to join the program, while the other ITNs predominantly included EU candidates given that most did not require a visa at any stage of the program. It is likely that in spite of its global nature, the MSCA-ITNs are more popular around European regions. Notably, irrespective of visa requirements, most students had prior international experience i.e., they had travelled abroad previously for various academic pursuits. Plausibly the previous international experiences prepared some ESRs better for the mobility related challenges and consequently exerted little impact on their academic performance, physical, psychological and overall well-being. Nonetheless, mobility related challenges did have moderate to severe impact on majority ESRs. Support for settling in the new country mostly came from host institutions and teams. This is perhaps one of the reasons that many ESRs from both the tinnitus-related and other ITNs opine that the ITN experience is largely dependent on the host institution. To participate in MSCA-ITNs it is mandatory to move to a new country. However, no settling allowance is provided to ESRs and about 90% of ESRs agreed that an initial allowance would be helpful.

Altogether, the perspectives of ESRs from other ITNs resonated with those of ESIT and TIN-ACT ESRs. The scientific and personal supervision was satisfactory for most ESRs from all ITNs, and the multidisciplinary ITN framework was well-received and appreciated as one of the noteworthy advantages in the ITN model. Notably, though 15–20% ESRs did not benefit from this multidisciplinary approach due to lack of common research interest, lack of other supervisors within their respective ITNs with the required subject matter expertise, and difficulties with host institutions preventing collaborations. It is somewhat surprising that in spite of the emphasis on interdisciplinary research in ITNs, there is still a lack of experts to seek support from or to collaborate with. A potential solution could be further enlarging the ITN network based on the research projects undertaken by the doctoral candidates so that adequate support can be provided. Although most doctoral candidates were satisfied with their collaborations, they were mostly limited to other ESRs or academic organisations within their own ITNs. This is an expected outcome given that with 15 ESRs and supervisors and multiple host institutions, the ITNs within themselves create a large enough network for multiple collaborations. However, it has to be noted that collaborations were comparatively lesser with industrial partners as well as with organisations outside the ITNs. Sometimes ITN-associated research projects are highly specific, pre-defined and time bound, therefore restricting opportunities to collaborate with researchers either within or outside the consortium.

Training schools and workshops are mandatory components of the ITN structure. Despite the consistently high ratings on different aspects of the training and workshops, ESRs recommended inclusion of relevant methodological and scientific topics, and a tailored approach depending on the background of ESRs and the stage of their PhD. Understandably, it is very difficult to accommodate the preferences of all trainees involved. All ESRs from tinnitus and non-tinnitus ITNs recommended involving the ESRs in the planning of training schools. This could increase the relevance of the training modules as well as customise them to individual trainee needs. Secondments, which are again another important aspect of MSCA-ITNs, place students in an organisation outside their host institution for a temporary period of time. Even though most of the ESRs from tinnitus-related ITNs had either an academic or industrial secondment, this did not influence their future career choices. On the contrary, all of the ESRs from other ITNs had a secondment and this experience influenced the career choices in approximately 40% of them. Therefore, the impact of secondments on future career choices is inconclusive from our present study. As some ESRs observed, the experience was too short to understand the workings of the sector and largely organisation-dependent. It is possible that while secondments help in providing experience, they are not particularly impactful for eventual career choices. Nonetheless, the majority of ESRs from all ITNs agreed that participation in an ITN itself was a prestigious opportunity in multidisciplinary research which contributed to professional and personal development and better career prospects.

The overall perception of the ITN experience was similar for ESRs from both tinnitus-related and non-related MSCA-ITNs. Development of personal and professional skills, international life experience, generous salary and research budget, and peer support made the experience worthwhile. However, a three-year PhD timeline as against the common four-year structure, in addition to the ITN and institute specific deliverables, bureaucratic documentations and travel for training and secondments, made the PhD journey within an ITN difficult and challenging. Some amount of flexibility in requirements plus having a more standardised EU framework across institutes could make the ITN experience comfortable and homogeneous for ESRs. Even though ESRs were satisfied with the overall ITN experience, it is interesting to note that most had no idea about the ITN structure in terms of travelling, networking and additional training in e.g., transferable skills, or how it was different or similar to a non-consortia-based PhD. It is therefore not surprising that the ITN experience in most cases surpassed the ESRs’ expectations.

Doing a PhD on an individual basis is a different kind of challenge than joining an ITN. Most individual PhD candidates have to manage research grants by themselves and only have a limited amount of travel/ research budget compared to ITN ESRs, which could make it more difficult to connect to the respective science community. Some ITNs also offer the possibility for individual PhDs to join ITN training schools and workshops in order to network and collaborate with ESRs working in the same field. In the following section, evaluation of ITNs from the perspectives of individual PhDs and in comparison to ITN ESRs are discussed.

Interestingly, half of the individual PhDs also did their PhD abroad. This finding can be explained by the fact that international research skills are fundamental if someone wants to pursue a career as a scientist. However, sometimes it could also be easier to go abroad since PIs and universities are particularly looking for international PhD-researchers. Individual PhDs more often felt fully integrated into their host team or institutions than ITN ESRs. A potential reason for that could be that ITN ESRs are seen as an independent entity with separate demands and problems compared to other doctoral students in the respective team. Further, half of the individual PhDs did their PhD locally. Perhaps lack of cultural and language barriers plus prior acquaintance with their future supervisor and their team contributed to a faster and easier integration process.

Compared to ITN ESRs, a lesser proportion of individual PhD candidates were satisfied with the scientific supervision. This difference might be related to the inherent structure of the ITNs, which enables the ESRs to contact many different individuals for scientific or personal advice. Whereas individual PhDs mostly rely on the scientific knowledge of the local supervision team. However, the quality of personal supervision was assessed similarly to ITN ESRs. The most obvious finding to emerge is the large disparity in satisfaction with respect to funding budget. Individual PhD candidates were less satisfied with their salary and research budget.

Since the main aim of the ITN is not the PhD itself, but rather conducting high-level research on relevant topics, ITN ESRs are employed as scientific staff with higher salary and a generous research budget. Additionally, EU-grants in general consist of a larger budget than purely national grants. Further and in contrast to ITN ESRs, the majority of individual PhDs stated that the local reimbursement process was not satisfactory. Better satisfaction for ITN ESRs could arise from the multilevel ITN structure, better regulation systems and strict time limits for EU-grants. However, it should be noted that the quality of reimbursement processes is highly institution-dependent.

It is somewhat surprising that individual PhD candidates evaluated ITN training schools and workshops lower than ITN-associated ESRs in all domains. This observed difference could have been caused by the expectation of the individual PhD candidates to have very high-level training given the prestigious EU framework of ITNs, or because the scientific content and level of training did not match with their PhD project and stage.

It would be expected that due to lower research/ travel budgets, individual PhD candidates probably do not have the opportunity to join as many conferences as ITN ESRs. Remarkably, not only did they attend more conferences, but also exhibited more active participation than ITN ESRs. Doing a PhD abroad, having several ITN-associated workshops and partnerships as well as the opportunity to collaborate with ITN colleagues, it might be possible that ITN ESRs feel they do not need to join a lot of conferences. In addition, potentially the combination of ITN and host institution associated deliverables might not allow the ESRs enough time for participation in external conferences.

Individual PhD candidates were satisfied with their collaborations, although most of them collaborated only with colleagues at their local host institutions. Similar to ITN ESRs, individual PhD students found the ITN workshops and training useful for their personal development and highlighted the opportunity to network during these events. Even though individual PhD students identified several advantages of ITNs, such as international experiences or network opportunities, the majority of them had the feeling that participating in an ITN would have made their PhD journey more challenging, whereas ITN ESRs think that joining an ITN made it easier for them. According to individual PhD students, ITNs have a larger number of requirements and activities, which could be time consuming and results in less time for the actual PhD, leading to unnecessary delays in PhD completion which can be a difficulty, particularly if it extends beyond the three-year funding period.

### Covid-19

Current doctoral students not only have to face the “usual” challenges of a PhD, but are also confronted with the present pandemic situation. During the year 2020, Covid-19 related restrictions affected the majority of PhD-projects, particularly those which required patient involvement and laboratory work. As a consequence, this could result in a loss of funding prior to the actual PhD completion, which induces anxiety and stress among ESRs. Adopting a more flexible approach by creating innovative and personalised solutions, such as reallocating research budget or switching face-to-face projects to online platforms, could be useful. Furthermore, expanding funding beyond the usual three-year period could enable ESRs to adequately complete their training and research projects in times of this global crisis.

### Strength and limitations

The biggest strength of this study is that the survey was developed by ESRs and therefore, addressed the domains and topics that were most pertinent for them. Further improvements were based on constructive inputs from experienced researchers. Being an anonymous online survey, it can be assumed that the participants were forthright with their responses, contributing to the quality of answers received. Although the survey was developed by ESRs from a tinnitus-related ITN, training networks from a variety of other scientific fields, including other health domains, technology or humanities, were reached out to participate. As a result, the scope of this investigation and implications of its results widened considerably.

The generalisability of these results is subject to certain limitations. For instance, the rather small sample size of individual PhD candidates is not adequate to make valid conclusions. Only nine out of 36 contacted ITNs participated in this survey and each of them are usually funding a limited number of 15 PhD candidates. Perhaps a larger number of respondents would strengthen our conclusions and provide a clearer picture of the benefits and disadvantages of participating in an MSCA-ITN for PhD studies. It might have been worthwhile to inquire about teaching/ supervision requirements of PhD candidates by their host institutions. However, this domain was not identified during our meeting with ESRs to assess the topics that need to be addressed.

## Conclusion

The MSCA-ITN framework provides a unique PhD opportunity that adds to doctoral students’ professional, personal and cultural experiences, along with a generous financial support. This study is the first attempt at evaluating the MSCA-ITN consortium-based PhD training and reports the ESR i.e., the student’s point of view of the experience. However, the ITNs have several other stakeholder and future studies evaluating their experiences, particularly those of ITN supervisors, could provide a more well-rounded perspective. To improve the ESR experience with regards to the host institution and supervision, future ITNs could provide additional mentoring training to supervisors and create a more unified structure to homogenise the ESR experiences. Future ITNs should also make the training and workshops tailored to fit student requirements, by involving students in planning of the training activities. The ITN-related collaborations could be further enhanced by nurturing collaborative projects with industry and organisations outside ITNs. Furthermore, introducing some flexibility in secondments, workshops and most importantly in the duration of PhD of MSCA-ITN could be helpful for ESRs. In conclusion, the MSCA-ITN provides a valuable experience for doctoral students that can be further enhanced by adding some modifications to the programme structure. The insights generated from this survey contribute vital implications for the successful implementation of future doctoral training programmes, either under the MSCA framework or other related schemes, highlighting the relevance and sustainability of our endeavour.

## Data availability

### Underlying data

Zenodo: Doctoral Studies as part of an Innovative Training Network (ITN): Early Stage Researcher (ESR) experiences - supplemental material & data. https://doi.org/10.5281/zenodo.4478894 ( Biswas *et al.*, 2021).

This project contains the following underlying data:

- raw_data_ITN_tinnitus.xlsx (survey results from PhDs as part of an ITN with a focus on tinnitus)
- raw_data_ITN_other.xlsx (survey results from PhDs associated to ITNs with another focus)
- raw_data_Individual_Phds.xlsx (survey results from PhDs not part of an ITN)

### Extended data

Zenodo: Doctoral Studies as part of an Innovative Training Network (ITN): Early Stage Researcher (ESR) experiences - supplemental material & data. https://doi.org/10.5281/zenodo.4478894 ( Biswas *et al.*, 2021).

This project contains the following extended data:

- Table1_ESIT Project Table.xlsx
- Table2_TIN-ACT Project Table.xlsx
- Table3_ITN_tinnitus.xlsx
- Table4_ITN_other.xlsx
- Table5_Individual PhDs.xlsx

Data are available under the terms of the Creative Commons Attribution 4.0 International license (CC-BY 4.0).
