# Supplementary material for: Doctoral Studies as part of an Innovative Training Network (ITN): Early Stage Researcher (ESR) experiences
Source: Open Res Eur. 2021 Sep 6;1:34. Originally published 2021 Apr 7. [Version 2] doi: 10.12688/openreseurope.13094.2 (PMC10446088; doi:10.12688/openreseurope.13094.2)
Supplement: Peer review history

# Peer review: Reviewer response for version 2

**DOI:** 10.21956/openreseurope.15096.r27544

**Published:** 2021-09-27

**Authors:** Sioux McKenna

**Affiliations:** 1 Centre for Postgraduate Studies, Rhodes University, Makhanda, South Africa

I think the authors have responded appropriately to the concerns raised by the reviewers.

Is the study design appropriate and does the work have academic merit?

Yes

Is the work clearly and accurately presented and does it cite the current literature?

Yes

If applicable, is the statistical analysis and its interpretation appropriate?

Yes

Are all the source data underlying the results available to ensure full reproducibility?

Yes

Are the conclusions drawn adequately supported by the results?

Yes

Are sufficient details of methods and analysis provided to allow replication by others?

Yes

Reviewer Expertise:

doctoral education, higher education studies

I confirm that I have read this submission and believe that I have an appropriate level of expertise to confirm that it is of an acceptable scientific standard.

# Peer review: Reviewer response for version 2

**DOI:** 10.21956/openreseurope.15096.r27545

**Published:** 2021-09-13

**Authors:** John Morrissey

**Affiliations:** 1 School of Microbiology, University College Cork, Cork, Ireland

I have no further specific comments. In the revised version, the authors improved the clarity of some sections as requested. All the comments I raised were responded to and although, I might still differ on some points, the responses are satisfactory. The quality of the presentation is high. I think this review makes a useful contribution to the discussion around ITNs.

Is the study design appropriate and does the work have academic merit?

Yes

Is the work clearly and accurately presented and does it cite the current literature?

Partly

If applicable, is the statistical analysis and its interpretation appropriate?

Yes

Are all the source data underlying the results available to ensure full reproducibility?

Yes

Are the conclusions drawn adequately supported by the results?

Partly

Are sufficient details of methods and analysis provided to allow replication by others?

Yes

Reviewer Expertise:

Research area is yeast biotechnology; have co-ordinated 1 ITN (FP7) and 1 EJD (H2020).

I confirm that I have read this submission and believe that I have an appropriate level of expertise to confirm that it is of an acceptable scientific standard.

# Peer review: Reviewer response for version 1

**DOI:** 10.21956/openreseurope.14164.r26847

**Published:** 2021-06-22

**Authors:** Sioux McKenna

**Affiliations:** 1 Centre for Postgraduate Studies, Rhodes University, Makhanda, South Africa

This article is potentially very interesting to a wide range of readers however the style hampers its ability to succeed. There are, in my view, two issues that need attention to ensure that this impressive study reaches the audience it should.

1. The incredibly high number of acronyms needs attention. The reader has to constantly work backwards and forward to make sense of the article.
2. The article is largely a report on a specific programme rather than a deliberation on the nature of doctoral education drawing on the analysis of this programme. The authors need to consider that their readers come from across the world and would have very little interest in this specific programme but would rather be interested in what this study can inform them for their own contexts. I think with some editing, this article can be very useful to readers such as myself who need to understand better the benefits and constraints of doctoral programmes such as this one.

Is the study design appropriate and does the work have academic merit?

Yes

Is the work clearly and accurately presented and does it cite the current literature?

Yes

If applicable, is the statistical analysis and its interpretation appropriate?

Yes

Are all the source data underlying the results available to ensure full reproducibility?

Yes

Are the conclusions drawn adequately supported by the results?

Yes

Are sufficient details of methods and analysis provided to allow replication by others?

Yes

Reviewer Expertise:

doctoral education, higher education studies

I confirm that I have read this submission and believe that I have an appropriate level of expertise to confirm that it is of an acceptable scientific standard, however I have significant reservations, as outlined above.

# Response

**Published:** 2021-07-21

**Authors:** Stefan Schoisswohl

**Affiliations:** University of Regensburg, Germany

Dear Sioux McKenna, Thank you very much for your positive and constructive feedback on our manuscript. Please find our answers to your raised points and comments in a point-by-point format below. Any changes to the manuscript are highlighted via track-change-mode as well as appended to each response.   

1. The incredibly high number of acronyms needs attention. The reader has to constantly work backwards and forward to make sense of the article.

Thank you for this suggestion. We tried to reduce the number of abbreviations in the revised manuscript. Acronyms like NRS, ECTS or CORDIS, which are only used once, were removed from the text. We hope these changes increased the readability of the manuscript. However, we would like to keep abbreviations such as ITN, ESR, EC, MSCA, which have been frequently repeated throughout the manuscript. For ease of understanding/reading, we added a section “abbreviations” to the end of the manuscript.  

1. The article is largely a report on a specific programme rather than a deliberation on the nature of doctoral education drawing on the analysis of this programme. The authors need to consider that their readers come from across the world and would have very little interest in this specific programme but would rather be interested in what this study can inform them for their own contexts. I think with some editing, this article can be very useful to readers such as myself who need to understand better the benefits and constraints of doctoral programmes such as this one.

Thank you for the comment. We agree that it would be interesting to a larger international reader base to know the benefits and constraints of doctoral training programme such as this and how it can be useful to them. We therefore, added the following paragraph: “The MSCA-ITN is a specific EU-based programme that provides a most suitable structure for creating international exchange opportunities and an innovative research environment for future researchers. This study critically evaluates the MSCA-ITN experience from an early career researchers’ perspective. It provides important insights on the benefits of a consortium-based PhD training model and also alludes to components that can be modified to make the experience even more fruitful for the trainees. The format of the MSCA-ITN can be replicated in other countries and world regions to provide doctoral students cutting-edge training, opportunities for personal and professional development and help them build a collaborative network right from the PhD stage. This study highlights important pros and cons of the consortium-based PhD model that various stakeholders of the MSCA-ITN and similar training programmes could take cognizance of in the planning and management stages.”

# Peer review: Reviewer response for version 1

**DOI:** 10.21956/openreseurope.14164.r26699

**Published:** 2021-04-20

**Authors:** John Morrissey

**Affiliations:** 1 School of Microbiology, University College Cork, Cork, Ireland

This is a useful contribution to the body of information on ITNs and it is valuable to get the views of ESRs  / PhD students while they are taking part. I think that the survey is sound and the data are presented well in figures. The analysis is based on the data and is balanced. The raw data are available on Zenodo. I have some suggestions that I think would improve the manuscript.

 In terms of other studies, it is mentioned that no study has looked at the experience of ESRs in ITNs but I am not convinced that this is completely correct. For example, the EU themselves conduct a large-scale survey (some of the questions from the EU survey were used in this study) and those data have been reported and published on-line. I think that the findings of that study versus the one here need to be considered, at the very least in the discussion section.

 There was also a paper that came from the QUANTFUNG ITN a few years back that is also relevant since that included ESR survey data and also covered some similar themes. That study should also be referenced and form part of the discussion here ^1^.

 I would define “ESR” early on and I would explain that in Horizon Europe these will be called “doctoral candidates” – I think that the acronym “ESR” will disappear from the lexicon quite quickly.

 Regarding MSCA-ITNs, there were (still are, I think), three types – the classic, the EJD and the ID. I think that all your data are from the classic type? The different styles should be mentioned in the introduction and it specified that this study focused on the classic. You could also mention this in the discussion since in Horizon Europe, there is more emphasis on the EJD and perhaps that will address (or make worse!) some of the issues that you found.

 I understand why it was performed like this but in terms of the purpose of the article and the study, I do not see value in separating the data of the two tinnitus-related ITNs (28 ESRs) and the other ITNS (58 ESRs). As far as I can see the results are very similar and presenting them separately duplicates a lot of text since analysis and explanation are similar. The messages are communicated much more clearly when they are there once with the full dataset – the disaggregated data are on Zenodo for whoever wants them and it is still possible to highlight any specific differences in the text.

 In the surveys, one of the options was that the EU/EC offered support (panels B and D). I am unsure what that actually means? Is in the Project Officer? In my experience, ESRs do not have much contact with the PO other than at the mid-term check/review. Indeed, most communication with the PO goes through the co-ordinator. Or is something else meant here?

 For instances where ages and similar are used to categorise ESRs, I think that median is more informative than mean.

 The data for the non-ITN PhDs are not shown graphically and it is quite heavy going to read all the numbers as text and then look to see how that compares to the ITN ESRs. Notwithstanding the lower numbers, if they data are worth including, they should be in the same format for direct comparison.

 The discussion covers the impact of secondments on career choice. I was not completely convinced that this was inconclusive – it seems around 40% in total said that it had an influence. But more importantly, I think that it should be recognised that most of these ESRs still had not completed their PhD when surveyed so it is also possible that there had not been time for the secondment to have had impact? Secondments and their analysis are complicated and I suspect that a deeper analysis is needed to get the nuances. For example, does “affected” the career choice mean that it caused an ESR to reconsider and change their choice? That is not really the aim – it is more to open ESRs to considering other sectors – thus "affecting" could also mean “considered and now understood the other sector but did not change my direction”? There could also be a difference depending on where the secondment was (e.g. maybe those already in industry had made up their mind).

 Wording of this sentence is awkward “ dissemination (WP5) while the remaining three (WP2, WP3 and WP4) address the research fields in which the projects are divided across.”

 There is a systematic typo where the space before the slash is missing (e.g. academic/ personal problems).

 The sentence should probably include the number of males (two, I think?) as well – it reads oddly “Nine individual PhD-students (six females; one preferred not to answer)”.

Is the study design appropriate and does the work have academic merit?

Yes

Is the work clearly and accurately presented and does it cite the current literature?

Partly

If applicable, is the statistical analysis and its interpretation appropriate?

Yes

Are all the source data underlying the results available to ensure full reproducibility?

Yes

Are the conclusions drawn adequately supported by the results?

Partly

Are sufficient details of methods and analysis provided to allow replication by others?

Yes

Reviewer Expertise:

Research area is yeast biotechnology; have co-ordinated 1 ITN (FP7) and 1 EJD (H2020).

I confirm that I have read this submission and believe that I have an appropriate level of expertise to confirm that it is of an acceptable scientific standard, however I have significant reservations, as outlined above.

## References

[1] : Vita activa in biotechnology: what we do with fungi and what fungi do with us. *Fungal Biology and Biotechnology* .2017;4(1) : 10.1186/s40694-017-0041-2 10.1186/s40694-017-0041-2 29276613PMC5738780

# Response

**Published:** 2021-07-21

**Authors:** Stefan Schoisswohl

**Affiliations:** University of Regensburg, Germany

Dear John Morrissey,

 Thank you very much for your positive and constructive feedback on our manuscript. Please find our answers to your raised points and comments in a point-by-point format below. Any changes to the manuscript are highlighted via track-change-mode as well as appended to each response.   

- In terms of other studies, it is mentioned that no study has looked at the experience of ESRs in ITNs but I am not convinced that this is completely correct. For example, the EU themselves conduct a large-scale survey (some of the questions from the EU survey were used in this study) and those data have been reported and published on-line. I think that the findings of that study versus the one here need to be considered, at the very least in the discussion section.

Thank you very much for your suggestion. Indeed the evaluation from the EU fits our current findings pretty well. We added the following section to the end of the discussion section. “Our current findings resonate with a recent MSCA fellow evaluation (26.9% ESRs from ITNs) from the European Commission. The evaluation likewise reports that most ESRs felt fully integrated into their host institution as well as received appropriate necessary support during their PhD. Further ITN-related training helped ESRs to not only improve scientific/ research knowledge but also provided support to work on various soft skills. The vast majority of ESRs declared that participation in an MSCA-ITN helped them to find employment. Similarly to our findings, 85.8% of ESRs rated the overall ITN experience as positive.”  

- There was also a paper that came from the QUANTFUNG ITN a few years back that is also relevant since that included ESR survey data and also covered some similar themes. That study should also be referenced and form part of the discussion here ^1^ .

Thank you very much for signposting us to this article. Indeed the survey on the ESRs from the QUANTFUNG ITN did cover some of our survey domains. We have added the following information in our discussion section comparing our study and results to the paper on Quantfung ITN. “To the best of our knowledge, this is the first attempt at gaining a student perspective on EU-funded consortia-based PhD programmes in various research disciplines using a detailed anonymous survey covering several aspects of the ESRs personal and professional development. A previous study was conducted by one MSCA-ITN (QuantFung ITN) where five ESRs of the QuantFung consortium were interviewed to understand the impact of this experience on their personal life and personal development (Weinhold et al., 2017).” “In the Quantfung ITN study, the members also appreciated the complementing effect of using different tools to work on the same topic (Weinhold et al., 2017).”  

- I would define “ESR” early on and I would explain that in Horizon Europe these will be called “doctoral candidates” – I think that the acronym “ESR” will disappear from the lexicon quite quickly. à NO – keep it

We would like to keep the term ESR in the manuscript. Since we are also using it in one of our other papers - Simoes et al., 2021. The abbreviation ESR is also already described pretty early in the introduction section.  

- Regarding MSCA-ITNs, there were (still are, I think), three types – the classic, the EJD and the ID. I think that all your data are from the classic type? The different styles should be mentioned in the introduction and it specified that this study focused on the classic. You could also mention this in the discussion since in Horizon Europe, there is more emphasis on the EJD and perhaps that will address (or make worse!) some of the issues that you found.

Thanks a lot for this comment, and we clarified by providing a short sentence in the introduction, stating that while three different MSCA ITNs exist, we focused on the standard kind: “At the time of writing, there exist three different subcategories of ITNs, a standard doctoral training network, as well as European Joint Doctorates and Industrial Doctorates. This work will be focusing on the standard category.”   

- I understand why it was performed like this but in terms of the purpose of the article and the study, I do not see value in separating the data of the two tinnitus-related ITNs (28 ESRs) and the other ITNS (58 ESRs). As far as I can see the results are very similar and presenting them separately duplicates a lot of text since analysis and explanation are similar. The messages are communicated much more clearly when they are there once with the full dataset – the disaggregated data are on Zenodo for whoever wants them and it is still possible to highlight any specific differences in the text.

This manuscript was initially developed as an endeavor to evaluate the experience of PhD students in the two tinnitus-related MSCA-ITNs, ESIT and TIN-ACT, by the ESRs participating in these two consortia. The evaluation of ESR experiences from various MSCA-ITNs from various disciplines was conducted to assess the agreement between the perspectives of ESR from the tinnitus-related ITNs. This also increased the generalizability of our results, and we could say that irrespective of the research discipline there was a general consensus in much of the ESR experiences.  

- In the surveys, one of the options was that the EU/EC offered support (panels B and D). I am unsure what that actually means? Is in the Project Officer? In my experience, ESRs do not have much contact with the PO other than at the mid-term check/review. Indeed, most communication with the PO goes through the co-ordinator. Or is something else meant here?

Thank you very much for pointing this out. Indeed, for ESRs, additionally to support from the consortium, at any point of time in the project (and indeed, also during/after the Midterm Meeting), it was possible to reach out to the Project Officer, an Ombudsperson, or as well National Contact Points, or dedicated departments at their respective host institutions. We refined the following sentence: “Figure 1A shows the impact of ITN-related mobility on the respondents’ academic performance, physical, psychological and overall well-being, with most reporting little to moderate impact. Host institutions and supervisors, ITN management team and EU project team (including Project Officer, Ombudsperson and National Contact Points) supported ESRs with accommodation, language courses and work permits (Figure 1B).”  

- For instances where ages and similar are used to categorise ESRs, I think that median is more informative than mean.

We think that the mean is appropriate to represent our findings. Interested readers can find the median values in the supplemental material.   

- The data for the non-ITN PhDs are not shown graphically and it is quite heavy going to read all the numbers as text and then look to see how that compares to the ITN ESRs. Notwithstanding the lower numbers, if they data are worth including, they should be in the same format for direct comparison. 

The aim of the current project was to evaluate the PhD-experience of ITN ESRs. Due to the small number of individual PhDs (N=9) associated with one of the ITNs, we consciously refrained from highlighting the results in figures.  

- The discussion covers the impact of secondments on career choice. I was not completely convinced that this was inconclusive – it seems around 40% in total said that it had an influence. But more importantly, I think that it should be recognised that most of these ESRs still had not completed their PhD when surveyed so it is also possible that there had not been time for the secondment to have had impact? Secondments and their analysis are complicated and I suspect that a deeper analysis is needed to get the nuances. For example, does “affected” the career choice mean that it caused an ESR to reconsider and change their choice? That is not really the aim – it is more to open ESRs to considering other sectors – thus "affecting" could also mean “considered and now understood the other sector but did not change my direction”? There could also be a difference depending on where the secondment was (e.g. maybe those already in industry had made up their mind).

Thank you very much for your suggestion. In light of your comment, we rephrased the sentence like this, highlighting the influence of secondments on career considerations: “For 37.3% respondents, these secondments helped in gaining perspective on the workings of different types of institutions, and influenced their interest in academia or industry for a potential work sector.”  

- Wording of this sentence is awkward “ dissemination (WP5) while the remaining three (WP2, WP3 and WP4) address the research fields in which the projects are divided across.”

Indeed. We changed the section as follows: “...which three address training (WP1), management (WP6) and dissemination (WP5). The remaining three WPs (WP2, WP3 and WP4) address the miscellaneous research fields of the different PhD-projects.”   

- There is a systematic typo where the space before the slash is missing (e.g. academic/ personal problems).

We checked the overall document and made the necessary corrections in the relevant places.  

- The sentence should probably include the number of males (two, I think?) as well – it reads oddly “Nine individual PhD-students (six females; one preferred not to answer)”.

To maintain consistency in reporting results, we did not mention the number of males here. However, we changed the sentence as follows: “Nine PhD-students (six females; one preferred not to answer) who were not MSCA-ITN funded but associated (by workshop participation, collaboration etc.) with the nine MSCA-ITN programmes, participated in the mITN-EQ.”
